# Is all bullying the same?

**DOI:** 10.1186/2049-3258-72-19

**Published:** 2014-06-09

**Authors:** Lihui Zhang, Lars Osberg, Shelley Phipps

**Affiliations:** 1 Johnson-Shoyama Graduate School of Public Policy, University of Regina, 110 – 2 Research Drive, Regina SK S4S 0A2, Canada; 2 Department of Economics, Dalhousie University, 6214 University Avenue, Halifax, NS B3H 3J5, Canada

**Keywords:** Bullying, Victim, Adolescent, Longitudinal

## Abstract

**Background:**

We ask whether verbal abuse, threats of violence and physical assault among Canadian youth have the same determinants and whether these determinants are the same for boys and girls. If these are different, the catch-all term “bullying” may mis-specify analysis of what are really different types of behavior.

**Methods:**

We analyze five cohorts of Canadian youth aged 12-15 from the National Longitudinal Survey of Children and Youth (NLSCY). There are 11475 observations in total. Pearson’s correlation coefficients and six different multivariate strategies are used.

**Results:**

There are many faces to bullying, in terms of its form and relative frequencies for boys versus girls. Although some characteristics of an adolescent are strong predictors of being subject to more than one type of bullying, some other characteristics are only correlated with specific types of bullying.

**Conclusions:**

The many faces of bullying, and their correlation with different factors, imply different policy interventions may be needed to address each issue effectively.

## Background

“Bullying” is an issue that has received much attention from academics [1-5], the media [6-10], government and international agencies [11-13], due to its prevalence and sometimes-serious negative consequences, such as victim mental health or even suicide. This paper examines the individual characteristics associated with being the victim of bullying among 5488 boys and 5987 girls aged 12-15 sampled in Canada’s National Longitudinal Survey of Children and Youth (NLSCY). As research [14,15] has documented, child and adolescent victims of bullying often do not report their experiences to police, parents, or teachers – hence this paper uses self-reported survey evidence on the experience of bullying.

However, being the victim of “bullying” is a much more ambiguous event than, for example, being hit by a car. The term “bullying” is often vaguely defined and subject to interpretation by respondents, which may be especially problematic when making cross-cultural comparisons [16]. The most prominent definition of bullying was proposed by Olweus: “a student is being bullied or victimized when he or she is exposed, repeatedly and over time, to negative actions on the part of one or more other students” [17]. This definition has been widely used in the scientific world. For example, Theriot et al state [18], that “the three key concepts that differentiate bullying (as defined by Olweus) from other forms of school violence and conflict are: (1) an intent to harm or upset another student, (2) the harmful behavior is done “repeatedly and over time”, and (3) the relationship between the bully (or bullies) and the victim(s) is characterized by an imbalance in power.” In the United Nations Children’s Fund (UNICEF) report cards on child well-being, bullying is seen as occurring when: “another student, or a group of students, say or do nasty and unpleasant things to him or her. It is also bullying when a student is teased repeatedly in a way he or she does not like or when he or she is deliberately left out of things. But it is not bullying when two students of about the same strength or power argue or fight. It is also not bullying when a student is teased in a friendly and playful way” [13]. Since intent, frequency and power imbalance are all difficult to define clearly and observe reliably, these definitions illustrate some of the complexities of research on bullying.

In this article, we address another level of complexity – the issue of whether all types of bullying behavior have the same determinants. Using the Canadian NLSCY, we ask whether researchers could be missing something by using the catch-all term of bullying to analyze different types of behavior. The NLSCY distinguishes verbal abuse, threat of violence, and physical assault as three different types of self-reported bullying. We ask: are they all just different manifestations of the same underlying phenomenon or are different personal characteristics associated with being the victim of each type of behavior?

## Methods

The data used in this research are the master-files of Cycles 3 to 8 of the National Longitudinal Survey of Children and Youth (NLSCY). The NLSCY, which started in 1994 and ended in 2008, is a longitudinal survey of factors that influence Canadian children’s social, emotional and behavioural development^a^ conducted biennially by Statistics Canada and sponsored by Human Resources and Social Development Canada. The target population was civilian, non-institutionalized residents living in Canada’s ten provinces. Excluded were residents of the Yukon, Nunavut and the Northwest Territories, people living on Indian reserves, full-time members of the Canadian Armed Forces and inmates of institutions. In each of these six cycles, Canadian youth were given a booklet comprising a battery of questions on their victimization experience in the past 12 months. To ensure confidentiality, the youth completed these questionnaires in private (away from parents and interviewers) and returned the booklet in a sealed envelope to the Statistics Canada interviewer. The main analytical sample in this research is five longitudinal cohorts of Canadian youth, who were 12 or 13 years old in 1998, 2000, 2002, 2004, and 2006, respectively. Each cohort might be interviewed a second time two years later when they were 14 or 15 years old, thus leading to an unbalanced two-year panel. In total, there are 11475 observations, with 5488 boys and 5987 girls, including repeated observations and siblings. Besides the youth self-completed components, the Person Most Knowledgeable (PMK) about the child (i.e. the mother in over 90% of the cases) also completed questionnaires containing a large array of information on the background of the child and/or the family, much of which will be used as explanatory variables in the multivariate analysis below.

In each cycle, each 12-15 year-old youth answers questions on three different types of victimization, verbal abuse, threat of violence, and physical assault at school or on a school bus.^b^ The wordings of the questions were:

• Verbal Abuse

In the past 12 months, how many times did someone say something personal about you that made you feel extremely uncomfortable: While at school or on a school bus?

• Threat of Violence

In the past 12 months, how many times did someone threaten to hurt you but did not actually hurt you: While at school or on a school bus?

• Physical Assault

In the past 12 months, how many times did someone physically attack or assault you: While at school or on a school bus?

The response categories were: 1) Never; 2) Once or twice; 3) 3 to 4 times; and 4) 5 times or more. One can think of the dependent variables as either categorical or as pseudo-continuous variables indicating the number of times a youth has been bullied. Specifically, to construct a pseudo-continuous estimate of frequency, we set the value of the dependent variable to 0 if the chosen answer is “Never”, 1.5 if “Once or twice”, 3.5 if “3 to 4 times”, and 5 if “5 times or more”.

Vulnerability to bullying can be plausibly argued to be determined along a number of dimensions. Our first set of independent variables refers to the personal characteristics of youth. For example, youth with disabilities may be “easy targets.” Or, children from visible minority groups may “stand out” as different and so also be potential targets. Also, children who are very small relative to their peers may seem vulnerable, so we include the youth’s height for age percentile constructed using the World Health Organization 2007 Reference. Finally, children who are overweight may be subject to bullying; thus, Body Mass Index (BMI) percentiles according to the guidelines of the Centre for Disease Control are included. To test the possibility that the “new kid” in a school may be subject to more bullying, we also use information provided in the NLSCY about whether the child changed school other than through natural grade progression and whether he/she changed residence in the past 2 years. Finally, child’s age is included to allow for the possibility that younger children experience more bullying.

We also include a set of family background variables to reflect the potential exposure of a child to socioeconomic disadvantage, which some past research suggests may increase vulnerability [19,20]. Family socioeconomic variables include family structure, parental age at child’s birth, parental immigrant status, education level of the PMK, and family equivalent household income. Family equivalent income is income from all sources before taxes but after transfers, divided by the square root of household size to take account of within-household economies of scale (the “Luxembourg Income Study Scale”).

Having siblings might mean that there are more chances for conflict at home, but may also mean youth can better develop social skills and therefore reduce victimization. Having an older sibling in the household may be protective of the younger sibling at school. We therefore include indicators both of whether or not the child has siblings in the household and of whether or not there is an older sibling.

Contextual variables describe the environment in which the child lives, or changes in that environment. Regions of residence and area population size are included since violence rates may differ in large urban areas compared to rural areas. Ideally, we would include an indicator for each province, but the discrete nature of the dependent variables and the small population size of some provinces necessitate aggregation to regions. We also control for attendance at a private school or public Catholic school, relative to public school since the kind of school the child attends is likely to be an important aspect of his/her environment.

The final sets of explanatory variables, though answered by the PMK, are more subjective in nature, but regarded as important in the literature. First, we have the PMK’s assessment of whether the child hangs out with peers who get in trouble and the PMK’s assessment of the quality of the neighbourhood environment. Specifically, the latter is a dummy variable coded 1 if the PMK strongly agrees that there are adults in neighbourhood that young people can look up to. Parental supervision is measured by the extent to which the PMK says she knows the child’s close friends.

To measure more subjective features of the home environment, we include a measure of PMK depression and of family functioning. The NLSCY provides a depression scale varying from 0 to 36, constructed from the PMK’s answers to a series of questions asking them how often they have felt or behaved this way during the past week, including, for example, “I did not feel like eating; my appetite was poor,” “I felt that I could not shake off the blues even with help from my friends,” “I had trouble keeping my mind on what I was doing,” or “I felt that everything I did was an effort”. Using a pooled sample of all families in the NLSCY 1998-2008, we consider a PMK to be depressed if his/her depression scale falls in the top 25th percentile. The NLSCY provides a family functioning score, ranging from 0 to 36, constructed from the PMK’s answers to a series of questions such as: “in times of crisis we can turn to one another for support,” “individuals within the family are accepted for what they are,” “we don’t get along well together” or “making decisions is hard for our family.” A higher score indicates greater family dysfunction. The score is standardized using a pooled sample of all families in the NLSCY 1998-2008.

Time spent by the child on computers is included to see if increased exposure to computer is connected to more exposure to bullying, possibly cyber-bullying. Unfortunately, time on computer is not available in cycles 3-4, i.e., 1998-2000, so a dummy variable is included to represent observations with missing values on computer time.

Finally, given recent media and policy attention to bullying, suggesting that bullying is a growing problem in Canada, all models include cohort dummies to test for changes in victimization for different cohorts, other things equal.

If being verbally abused, threatened with violence or having experienced violence are really just different aspects of the same underlying phenomenon, one would expect all three types of experiences to be highly correlated and to occur with somewhat similar frequency. To examine this, we start with descriptive statistics on the mean number of times bullied in the past 12 months and estimates of Pearson’s correlation coefficients [21,22] between different types of bullying. Both the means and the correlation coefficients are weighted using longitudinal sampling weights provided in the NLSCY.

We then estimate the multivariate model, as described by Eq. (1), using six different econometric techniques: i) ordinary least squares (OLS) regressions; ii) ordered probit regressions; iii) interval regressions; iv) random-effects regressions; v) random-effects interval regressions; and vi) Hausman-Taylor regressions [23,24]. Although researchers may agree on the general specification of Eq. (1), they may differ in their choice of econometric technique and we want to know if our conclusions regarding the significance of particular variables are robust to this choice.

In Eq. (1), y_it_ indicates a youth’s experience of being the victim of a particular type of bullying behaviour in the past 12 months, X_it_ is a set of time-varying individual characteristics (e.g., family structure, family income, etc.), W_it_ is a set of time-invariant individual characteristics (e.g., race, immigration status, etc.), α_i_ is a random individual-specific effect, and ϵ _it_ is an idiosyncratic error.

(1) $$\begin{array}{ll} y_{\mathrm{it}} & =X{'}_{\mathrm{it}}\beta +W{'}_{\mathrm{it}}\gamma +\alpha_{i}+\in_{\mathrm{it}},t\\ & =1,2,3;i=1,2,\ldots ,N \end{array}$$

Let M′_it_ = [X′_it_, W′_it_]. As a starting point, OLS regressions may be used to estimate this model when y_it_ is thought of as pseudo-continuous, that is, the number of times, where the assumption is that α_i_ + ϵ _it_ is i.i.d. If y_it_ is instead seen as categorical, as represented by the four possible responses to the victimization questions, ordered probit regressions are the appropriate estimation technique. Alternatively, since each response can also be thought of as an interval, interval regressions can also be estimated, where y_it_ then includes two variables, one of which takes the lower bound value and the other takes the upper bound value of each interval. That is, for interval regressions, each regression has two dependent variables, y^1^_it_ and y^2^_it_. For example, if a youth was bullied 3 to 4 times, then y^1^_it_ = 3 and y^2^_it_ = 4. If 5 times or more, then y^1^_it_ =5 and y^2^_it_ is set to missing value.

Another candidate for estimating Eq. (1) is the random-effects model, which assumes that M′_it_ is not correlated with ϵ _it_ or α_i_, though α_i_ + ϵ _it_ is not i.i.d. This may be true as with panel data repeated observations for the same individual across time are likely to be correlated. Both the conventional random-effects regressions and the random-effects interval regressions are estimated.

The assumption that M′_it_ is uncorrelated with the individual-specific effect, α_i_, may be too strong. That is, one or more regressors in M′_it_ may be endogenous. If individuals differ from each other in unobserved ways and such individual effects are correlated with some regressors but largely fixed over time, for example, personality types, then a fixed-effects model may be more appropriate. By differencing appropriately, the fixed-effects model can consistently estimate the coefficients for the time-varying regressors, β, though the coefficients for the time-invariant regressors, γ, cannot be identified as they are eliminated by differencing. This is unfortunate because in many occasions it is the time-invariant regressors (e.g. visible minority status), which are of interest to the researcher. The Hausman-Taylor model provides an alternative to fixed-effects regressions if a subset of M′_it_ is uncorrelated with α_i_. Thus, Eq. (1) can be rewritten as Eq. (2), where X′_it_ = [X′_1it_, X′_2it_] and W′_it_ = [W′_1it_, W′_2it_]. X′_1it_ and W′_1it_ are assumed to be exogenous, whereas X′_2it_ and W′_2it_ are endogenous, i.e., correlated with α_i_. If this assumption holds, then, with appropriate random-effects transformation, data from other periods may be used as instruments to help obtain a consistent estimator for γ.

(2) $$\begin{array}{ll} y_{\mathrm{it}} & =X{'}_{1\mathrm{it}}\beta_{1}+X{'}_{2\mathrm{it}}\beta_{2}+W{'}_{1\mathrm{it}}\gamma_{1}+W{'}_{2\mathrm{it}}\gamma_{1}+\alpha_{i}+\in_{\mathrm{it}},\\ \;t & =1,2,3;i=1,2,\ldots ,N \end{array}$$

These multivariate regressions are unweighted, as the statistical software used, STATA, does not allow probability sampling weights for some of the estimations, for example, Hausman-Taylor regressions, random-effects regressions, and random-effects interval regressions.

## Results and discussion

Table 1 reports the means and standard errors for the number of times a 12-15 year-old adolescent is bullied at school or on a school bus in the past 12 months. First, for both boys and girls, verbal abuse occurs more frequently than threat of violence, which in turn is more frequent than physical assault. But the relative frequency of types of bullying is not the same by gender – girls are more likely to be subject to verbal abuse, while boys are more likely to be subject to threat of violence and physical assault. These simple statistics suggest that using catch-all questions on bullying is likely to mask the different frequencies of types of bullying for different genders. The high prevalence of verbal abuse among both genders and its potential harm may be particularly relevant today given the expediency and anonymity allowed for by the readily available internet [25,26].

**Table 1:** Number of times bullied at school or on a school bus in the past 12 months

	**Verbal abuse**		**Threat of violence**		**Physical assault**	
	**Boys**	**Girls**	**Boys**	**Girls**	**Boys**	**Girls**
Mean	1.12	1.23	0.74	0.34	0.28	0.10
Standard error	0.03	0.03	0.03	0.02	0.02	0.01
Number of observations	5488	5987	5488	5987	5488	5987

Data source: Cycles 3-8 of the NLSCY.

Table 2 shows the Pearson’s correlation coefficients across different types of bullying. All correlation coefficients are highly statistically significant, suggesting that adolescents who are subject to one type of bullying are also at higher risk of another type of bullying. However, the correlations between verbal abuse and the other two types of bullying are smaller than the correlations between threat of violence and physical assault. Notably, the correlation between being the victim of physical assault and being the victim of verbal abuse is positive, but not particularly high. This suggests that verbal and physical violence are unlikely to be different manifestations of the same underlying phenomenon but more likely are different phenomena.

**Table 2:** Pearson’s correlation between types of bullying at school or on a school bus

	**Boys**			**Girls**		
	**Verbal abuse**	**Threat of violence**	**Physical assault**	**Verbal abuse**	**Threat of violence**	**Physical assault**
Verbal abuse	1.00			1.00		
Threat of violence	0.35*	1.00		0.34*	1.00	
Physical assault	0.27*	0.52*	1.00	0.23*	0.36*	1.00

Data source: Cycles 3-8 of the NLSCY.

*indicates that the correlation coefficient is statistically significant at 1% level.

Table 3 summarizes the multivariate regression results on being bullied at school or on a school bus for three different types of bullying. As discussed above, six different econometric specifications were estimated, corresponding to different plausible researcher choices of statistical methodology. For succinctness, Table 3 counts the number of times a regressor is statistically significant at 5% level or better and the sign of the coefficient. In a few instances, one specification produces statistically significant results, but the others do not. More often, the data tell a qualitatively consistent story across the different specifications, with specific variables being either statistically insignificant in all specifications or statistically significant in all, or almost all, specifications.

**Table 3:** Times a correlate significant at 5% level, bullied at school or on a school bus

	**Boys**			**Girls**		
	**Verbal abuse**	**Threat of violence**	**Physical assault**	**Verbal abuse**	**Threat of violence**	**Physical assault**
Child has a disability		6; +	6; +			6; +
Child is aboriginal or black			1; +			
Child is Asian						3; +
Child’s height for age below 15th percentile	6; +					
Child’s height for age above 95th percentile						
BMI below 25th percentile		1; +				
BMI 85th percentile or above				5; +	5; +	
Age of child		1; -	6; -	6; +	6; -	6; -
Lone-parent family						
Stepfamily					6; +	4; +
Either parent a teenager at child’s birth						
Either parent an immigrant				6; -		
PMK has post-secondary degree						
Log of household equivalent income (2003 constant dollar)					3; -	
Child has a sibling						
Child has an older sibling						
Atlantic Canada						
Quebec		6; -	6; -		6; -	6; -
Prairies						
British Columbia						
Rural area						
Urban area, population 100,000 to 499,999	5; -			1; -		6; +
Urban area, population 500,000 or over						
Child attends a private school	6; +				1; -	
Child attends a public Catholic school						6; -
Changed school other than natural grade progression in the past 2 years			5; +	6; -		
Changed usual place of residence in the past 2 years	1; +				2; +	
Child hangs out with trouble kids		5; +	5; +	5; +	6; +	6; +
Strongly agree that there are adults in neighborhood that young people can look up to		1; +				
PMK knows few or half of child’s close friends					6; +	2; +
PMK knows all of child’s close friends		1; -		2; -	1; -	5; -
PMK depression score above 75th percentile						
Standardized family functioning score		5; +	1; +			
Hours per day child spends on computer	1; -	5; +	5; +	6; +	6; +	6; +
Hours per day child spends on computer not available	4; +			5; +		
Cohort 2000	6; -			6; -		
Cohort 2002	6; -			6; -		
Cohort 2004	6; -			6; -		
Cohort 2006	6; -			6; -		
Number of observations	5488			5987		

Data source: Cycles 3-8 of the NLSCY.

This table summarizes the regression results from six different econometric techniques: i) OLS regressions; ii) ordered probit regressions; iii) interval regressions; iv) random-effects regressions; v) random-effects interval regressions; and vi) Hausman-Taylor regressions.

Numbers in each cell indicate the number of times a regressor is statistically significant at 5% level or lower across the six techniques, followed by the sign of the coefficients.

The following explanatory variables are treated as endogenous in the Hausman-Taylor regressions: BMI below 25th percentile, BMI 85th percentile or above, changed school other than natural grade progression in the past 2 years, changed usual place of residence in the past 2 years, child hangs out with trouble kids, PMK knows few or half of child’s close friends, PMK knows all of child’s close friends, and hours per day child spends on computer.

If the determinants of all different types of bullying are the same, then there is little lost by aggregating the three types of bullying identified in the NLSCY into a catch-all measure of “bullying”. In some instances, this appears to be a reasonable procedure – Table 3 indicates, for example, that sibling status, lone parent family, PMK depression and residence in Atlantic Canada, the Prairies or British Columbia have the same level of correlation (i.e. insignificance) with all three types of bullying in a school environment.

However, consistent with the significant correlation coefficients shown in Table 2, some other characteristics predict differing chances of victimization across different types, for example, disability, peer influence, residing in Quebec, and computer time for both boys and girls, and, overweight status, family structure, and parental supervision for girls. As well, some characteristics also appear to be connected to one type of bullying but not others. For example, boys whose height for age is below the 15th percentile are verbally abused at school but they do not appear more likely to be threatened or physically attacked. Boys who attend private schools are subject to verbal abuse, but not threat of violence or physical assault. Change of school is associated with more physical assault at school for boys and less verbal abuse at school for girls.

Although some characteristics of children are similarly correlated with being the victim of all three different types of bullying, this is not always the case. For example, whatever estimation technique is used Table 3 indicates that there is something about the Quebec school environment that lessens the likelihood that a child will be the victim of physical assault or the threat of violence – but all six estimation techniques also concur that Quebec schools are not significantly different from those in other Canadian regions in the likelihood of a child experiencing verbal abuse. Evidently, one cannot necessarily assume that success in lessening some types of bullying will predict reductions in all other types of bullying behavior, which implies that different types of anti-bullying interventions may have different types of impacts.

## Conclusions

This paper sets out to answer the following research question: are verbal abuse, threat of violence, and physical assault different manifestations of the same underlying phenomenon or are different personal characteristics associated with being the victim of each type of behavior? In addition to differentiating different types of bullying for both boys and girls, this research, using self-reported confidential data representative of 12-15 year-old Canadian youth, provides a useful complement to bullying victimization reported by police, parents, and teachers, who may only be partially aware of the bullying experiences by young people. The empirical analysis in this paper indicates that:

• Bullying is not all the same. Rather, there are many faces to bullying, in terms of its form and relative frequencies for boys versus girls.

• Though some characteristics and background of an adolescent are strong predictors of being subject to more than one type of bullying, some other characteristics and background are connected to specific types of bullying.

The lesson we therefore take is that information gathering and analysis on bullying should not be limited to catch-all aggregations of all types of bullying behavior. While there are some issues for which this may be appropriate, it is in general desirable to be cognizant of the many faces of bullying, which not only are correlated with potentially different factors and amenable to different interventions, but also may very well have different consequences for the victims, thus, potentially different policy implications. Recognizing this complexity might be particularly important for researchers and policy makers, as they attempt to undertand the dynamics and determinants of bullying through comparisons of bullying across space, time, demographic, socio-economic, and cultural groups. The catch-all term of bullying will provide misleading results, if what is included in bullying differs along the above-mentioned dimensions. Researchers tasked with revisiting the methodologies for large-scale international studies, such as the Health Behaviour in School-aged Children study, might wish to take this into consideration [27].

### Endnotes

^a^Though the NLSCY, spanning from 1994 to 2008, provides the potential for the examination of time trends, this is not the focus of this paper.

^b^The working paper version of this article (available upon request from the corresponding author) included results for bullying elsewhere (including at home). Professor Michal Molcho, the reviewer for this paper, commented that: “While bullying can occur in the home, incidents of verbal and physical abuse, as well as threats, coming from the carer (parent, for example), fall under child abuse rather than bullying. Child abuse has different, and more severe, consequences”. We concur with Professor Molcho’s concern, thus have removed these results from the paper.

### Consent

Written informed consent was obtained from the patient’s guardian/parent/next of kin by Statistics Canada for the publication of this report and any accompanying images.

## Abbreviations

BMI: Body mass index; NLSCY: National longitudinal survey of children and youth; OLS: Ordinary least squares; PMK: Person most knowledgeable; UNICEF: United nations children’s fund.

## Competing interests

The authors declare that they have no competing interests.

## Authors’ contributions

LZ performed the statistical analysis. All authors conceived of the study, participated in the design of the study, and worked together to draft the manuscript. All authors read and approved the final manuscript.
